# New species of *Polystomoides* (Monogenoidea: Polystomatidae) parasitizing the urinary bladder of a freshwater turtle in Brazil

**DOI:** 10.1590/S1984-29612023045

**Published:** 2023-07-24

**Authors:** Julia Somavilla Lignon, Simone Chinicz Cohen, Marcia Cristina Nascimento Justo, Louis Du Preez, Carine Glaucia Comarella, Rogerio Akio Nishimaru, Paulo Vinicius Abbade Moreira Souza, Michelli Westphal de Ataíde, Daniel Curvellho de Mendonça Müller, Maurício Veloso Brun, Silvia Gonzalez Monteiro

**Affiliations:** 1 Laboratório de Parasitologia Veterinária, Departamento de Microbiologia e Parasitologia, Universidade Federal de Santa Maria - UFSM, Santa Maria, RS, Brasil; 2 Laboratório de Helmintos Parasitos de Peixes, Instituto Oswaldo Cruz, Fundação Oswaldo Cruz - FIOCRUZ, Rio de Janeiro, RJ, Brasil; 3 Unit for Environmental Sciences and Management, North-West University, Potchefstroom, South Africa; 4 South African Institute for Aquatic Biodiversity, Makhanda, South Africa; 5 Endosolutions Vet, Campinas, SP, Brasil; 6 Faculdade de Medicina Veterinária e Zootecnia, Universidade Estadual Paulista “Júlio de Mesquita Filho” - UNESP, Jaboticabal, SP, Brasil; 7 Universidade de Passo Fundo - UPF, Passo Fundo, RS, Brasil; 8 Departamento de Clínica de Pequenos Animais, Universidade Federal de Santa Maria - UFSM, Santa Maria, RS, Brasil

**Keywords:** Platyhelminthes, chelonians, Rio Grande do Sul, Trachemys dorbigni, Platelmintos, quelônios, Rio Grande do Sul, Trachemys dorbigni

## Abstract

*Trachemys dorbigni* is the most abundant freshwater turtle species in Rio Grande do Sul, southern Brazil. Chelonians are known to host a wide variety of pathogens, including viruses, bacteria, hemoparasites and helminths. Among these, nine genera of polystomatid flatworms (Monogenoidea; Polystomatidae) infect freshwater turtles: *Apaloneotrema*, *Aussietrema, Fornixtrema*, *Manotrema*, *Pleurodirotrema*, *Polystomoidella, Polystomoides, Uropolystomoides* and *Uteropolystomoides*. However, little is known about the biology of these parasites in the Neotropical Realm. Through investigative cystoscopy, specimens of Polystomatidae were located inside the urinary bladder of the host *T. dorbigni*. Retrieved specimens were fixed and stained whole mounts prepared for taxonomic identification. In the present paper, a new species of *Polystomoides* (Monogenoidea: Polystomatidae) parasitizing the urinary bladder of a freshwater turtle of the species *T. dorbigni* in Brazil is described. *Polystomoides santamariensis* n. sp. differs from the congeneric species on the length of the genital spines, which are longer. Given the enormous diversity of freshwater turtles around the world, it is likely that a large number of chelonian polystomatids are still unknown.

## Resumo

*Trachemys dorbigni* é a espécie de tartaruga de água doce mais abundante do Rio Grande do Sul, no Sul do Brasil. Sabe-se que os quelônios são hospedeiros de uma grande variedade de patógenos, incluindo vírus, bactérias, hemoparasitos e helmintos. Dentre estes, oito gêneros de Polystomatidae (Platyhelminthes: Monogenoidea) infectam tartarugas de água doce: *Apaloneotrema*, *Aussietrema, Fornixtrema*, *Manotrema*, *Pleurodirotrema*, *Polystomoidella, Polystomoides, Uropolystomoides* e *Uteropolystomoides*. No entanto, pouco ainda se sabe sobre a biologia destes parasitos na Região Neotropical. Durante uma citoscopia investigativa, foram observados espécimes de Polystomatidae localizados no interior da bexiga urinária de *T. dorbigni*. Os espécimes coletados foram fixados e corados para identificação taxonômica. No presente artigo, uma nova espécie de *Polystomoides* (Monogenoidea: Polystomatidae) parasitando a bexiga urinária de tartaruga de água doce da espécie *T. dorbigni* no Brasil é descrita. *Polystomoides santamariensis* n. sp. difere das demais espécies no tamanho dos espinhos genitais, que são maiores. Devido a enorme diversidade de tartarugas de água doce ao redor do mundo, é provável que um grande número de polistomatídeos de quelônios ainda seja desconhecido.

## Introduction

With 795 of the 11,733 known reptiles species of the world from Brazil, it is the country with the third-largest diversity of reptiles (Costa & Bérnils, 2018; Uetz et al., 2023). The order Testudines comprises turtles, tortoises and terrapins (freshwater turtles) and is composed of two suborders: Pleurodira and Cryptodira. The first has distribution only in the southern hemisphere, across the Australian, Ethiopian and Neotropical Realms, while Cryptodira has the largest number of living species, with distribution on all continents, with the exception of Australia and Antarctica (Van Dijk et al., 2014; Rhodin et al., 2021; Du Preez et al., 2022). In Brazil, 36 species of Testudines have been described (Costa & Bérnils, 2018) and in the state of Rio Grande do Sul, there are 11 chelonian species, among which five are marine and six are freshwater species (Lema & Ferreira, 1990). Among the latter, *Trachemys dorbigni* Duméril & Bibron, 1835, is the most abundant (Bujes et al., 2011). In Brazil, it occurs naturally in Rio Grande do Sul and Santa Catarina, and it also inhabits other countries such as Argentina and Uruguay (Rhodin et al., 2021).

Species of *Trachemys* Agassiz, 1857 present the widest geographical distribution among New World chelonians, such that their range extends from the United States to Argentina (Seidel, 2002). This genus comprises 15 species and, in addition to *Trachemys dorbigni,* in South America *Trachemys callirostris* Gray, 1856, is found in Colombia and Venezuela and *Trachemys adiutrix* Vanzolini, 1995, in Brazil (Van Dijk et al., 2014).

Freshwater turtles are known to host a wide variety of pathogens that belong to all major groups of parasites (e.g. viruses, bacteria, hemoparasites and helminths). Among these, Polystomatidae (Platyhelminthes: Monogenoidea) is the largest family of monogenoideans and comprises 32 genera, among which *Apaloneotrema* Du Preez & Verneau, 2020, *Aussietrema* Du Preez & Verneau, 2020, *Fornixtrema* Du Preez & Verneau, 2020, *Manotrema* Du Preez, Domingues & Verneau, 2022, *Pleurodirotrema* Du Preez, Domingues & Verneau, 2022, *Polystomoidella* Price, 1939, *Polystomoides* Ward, 1917, *Uropolystomoides* Tinsley & Tinsley, 2016 and *Uteropolystomoides* Tinsley, 2017 infect freshwater turtles (Morrison & Du Preez, 2011; Chaabane et al., 2022; Du Preez et al., 2022).

At present from the Neotropical realm three species are known from the conjunctival sacs of their chelonian hosts namely *Fornixtrema fentoni* (Platt, 2000) from *Kinosternon leucostomum* (Duméril & Duméril, 1851) and *Rhinoclemmys pulcherrima* (Gray, 1856), *Fornixtrema guianense* (Du Preez et al., 2017) from *Rhinoclemmys punctularia* (Daudin, 1801) and *Fornixtrema scorpioides* (Du Preez et al., 2017) from *Kinosternon scorpioides* (Linnaeus, 1766).

From the oral region of their chelonian hosts currently are known *Manotrema brasiliensis* (Vieira et al., 2008) from *Hydromedusa maximiliani* (Mikan, 1820) and *Phrynops geoffroanus* (Schweigger, 1814), *Manotrema fuquesi* (Mané-Garzon & Gil, 1962) and *Manotrema uruguayensis* (Mané-Garzon & Gil, 1961) from *Phrynops hilarii* (Duméril & Bibron, 1835), *Polystomoides magdalenensis* Lenis & Garcia-Prieto, 2009 from *Trachemys callirostris callirostris* (Gray, 1856) and *Polystomoides rohdei* Mané-Garzon & Holcman-Spector, 1968 from *Trachemys dorbigni* (=*Pseudemys dorbigni*).

From the urinary bladder of their chelonian hosts are known *Polystomoides cayensis* (Du Preez et al., 2017) from *Rhinoclemmys punctularia* and *Polystomoides domitilae* (Caballero, 1938) Price, 1939 from *Chrysemys ornata* (= *Trachemys scripta ornata*).

Through investigative cystoscopy after video-assisted ovariosalpingectomy performed on an individual of the species *T. dorbigni*, specimens of Polystomatidae were found. In the present paper, a new species of *Polystomoides* (Monogenoidea: Polystomatidae) parasitizing the urinary bladder of this freshwater turtle in Brazil is described.

## Material and Methods

One specimen of *T. dorbigni* (D'Orbigny's slider or “Tartaruga-tigre-d'agua”) was subjected to investigative cystoscopy after video-assisted ovariosalpingectomy in the Veterinary Hospital of the Federal University of Santa Maria (UFSM). This animal came from the São Braz conservation breeding station (“Criadouro Conservacionista São Braz”), located in Santa Maria, Rio Grande do Sul, Brazil (29º 41′ 03” S; 53º 48′ 25” W). Elective castration was performed on this individual as a population control measure.

During the cystoscopy, using a 30° scope Karl Storz^TM^, Tuttlingen, Germany (1.9-2.0 mm) inside in a working sheath (9.6 Fr.) and semi-rigid forceps (Karl Storz^TM^, Tuttlingen, Germany), three helminth specimens that were found attached to the urinary bladder were collected. These were sent to the Veterinary Parasitology Laboratory of UFSM for identification at the species level, and subsequently to the Laboratory for Helminth Parasites of Fish, Oswaldo Cruz Institute, Oswaldo Cruz Foundation. The samples were fixed in 70% alcohol and stored. Specimens were stained with Langeron’s alcoholic acid carmine, dehydrated in an ethyl alcohol series, cleared in beechwood creosote and mounted in Canada balsam as permanent slides (Eiras et al., 2006). Measurements are presented in micrometers, unless otherwise stated, as range values followed by mean values, when more than two, and the number of structures measured, where applicable, in parentheses. Specimens were illustrated with the aid of a camera lucida coupled to a Zeiss Axioskop light microscope. Light microphotographs were made with the use of the ZEN 2 software (Blue edition) ^®^; Carl Zeiss Microscopy, 2011. The authorship of the taxa followed the recommendation of Article 50.1 of the International Code of Zoological Nomenclature (ICZN), which deals with the identity of the authors. The holotype and paratypes were deposited in the Helminthological Collection of the Oswaldo Cruz Institute (“Coleção Helmintológica do Instituto Oswaldo Cruz”, CHIOC), Rio de Janeiro, Brazil.

## Results

Class Monogenoidea Bychowsky, 1937

Order Polystomatidea Lebedev, 1988

Family Polystomatidae Gamble, 1896

*Polystomoides* Ward, 1917

**Figure 1:**
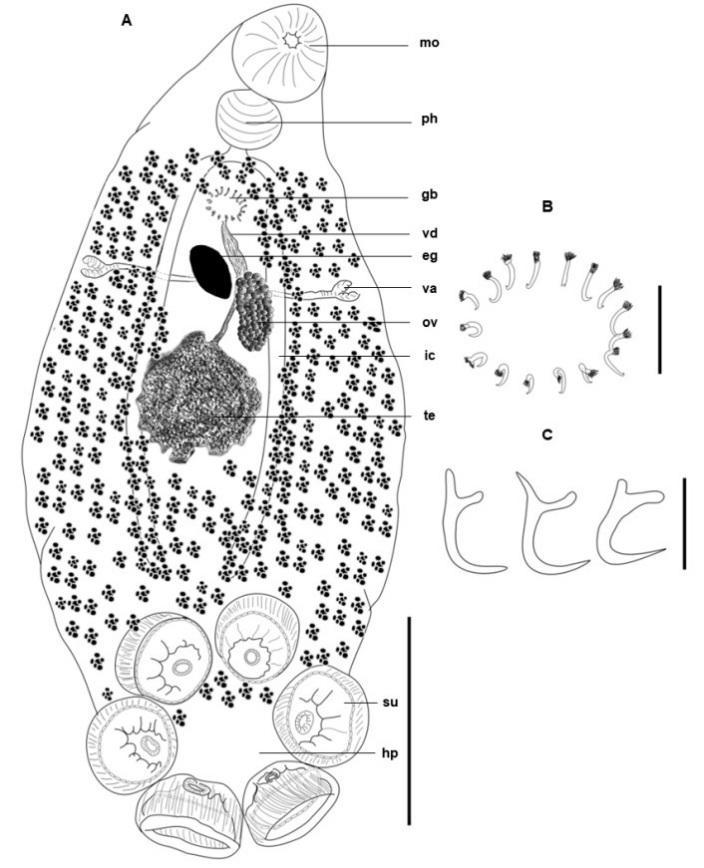
*Polystomoides santamariensis* n. sp. Holotype. A - Ventral view. Bar= 1mm; B - Genital spines. Bar= 100 μm; C - Marginal hooklets. Bar= 10 μm. Abbreviations: mo, mouth; ph, pharynx; gb, genital bulb; vd, vas deferens; eg, egg; va, vagina; ov, ovary; ic, intestinal cecum; te, testis; su, sucker; hp, haptor.

**Figure 2:**
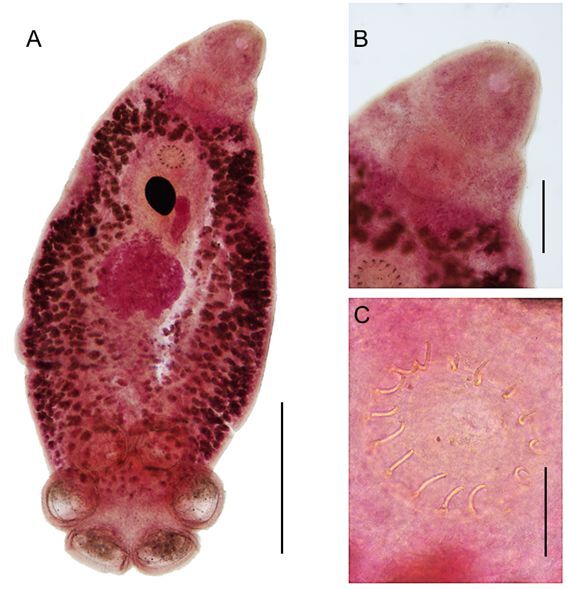
*Polystomoides santamariensis* n. sp. A - Total, ventral view. Bar= 1mm; B - Anterior region, showing mouth and pharynx. Bar= 300 μm; C - Genital bulb with 16 spines. Bar= 100 μm.

**Figure 3:**
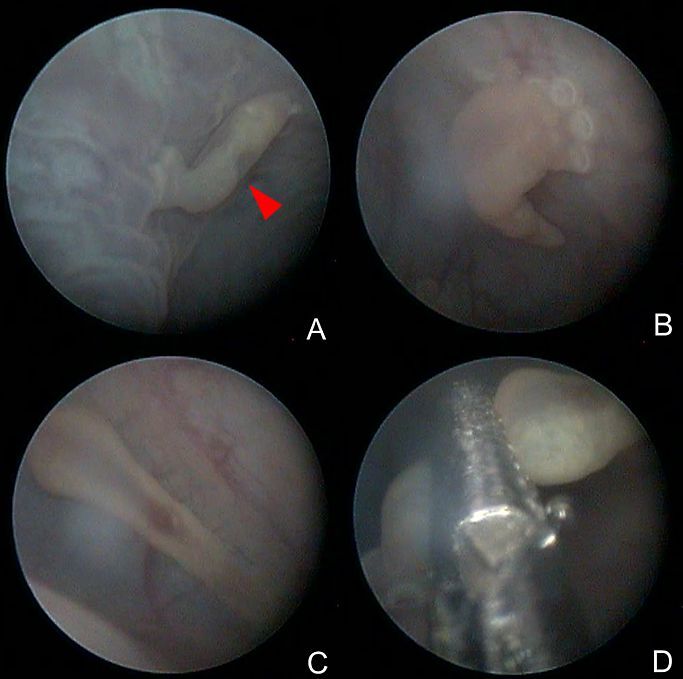
Specimens of *Polystomoides santamariensis* n. sp. in host’s urinary bladder (*Trachemys dorbigni*). A - Parasite fixed in mucosa (arrow); B - View of its suckers; C - Parasite stretching to move; D - Specimen collected using semi-rigid forceps.

*Polystomoides santamariensis* Lignon, Cohen, Justo, Du Preez & Monteiro n. sp. (Figures 1-3)

**Host:***Trachemys dorbigni* (Duméril & Bibron, 1835) (adult female)

**Site:** Urinary bladder

**Locality:** Santa Maria, Rio Grande do Sul, Brazil (29º 41′ 03” S; 53º 48′ 25” W).

**Type-material:** Holotype CHIOC 39994 a, Paratypes CHIOC 39994 b, c.

**Etymology:** The specific name refers to the locality, Santa Maria.

**Description** (based on 3 sexually mature specimens stained with acid carmine): Body elongated and ellipsoid, 3,000-4,350 (3,742) long by 1,500-1,675 (1,600) wide and 1,400 at vaginae level (Figures 1A, 2A, 3A). Mouth subterminal, surrounded by a false oral sucker, 410-470 (443) long by 410-450 (423) wide; Pharynx muscular, subspherical, 235-265 (255) long by 295-330 (310) wide (Figures 1A, 2A, B); esophagus short and intestine bifurcates into two intestinal caeca, non-confluent posteriorly to gonads, lacking diverticula, extending to posterior region of trunk, not reaching the haptor region. Intercaecal gonads, testis posterior to ovary. Testis single, spherical with poorly developed lobes, mid-ventral, posterior to ovary, 530-600 (560) long by 610-700 (643) wide. Seminal vesicle a dilatation of vas deferens, sigmoid, crossing midline, followed by genital bulb (Figures 1A, 2A). Genital bulb 155 and 175 long by 160 and 200 wide, presenting 16 genital spines, 37-42 (40; n = 8) long (Figures 1B, 2C). Ovary sinistral, elongated, 240 and 320 long by 100 and 110 wide. Two vaginae present, located marginally, at level of middle of body proper, intervaginal distance of 85.7% of the body width at the level of the vaginae, located at 1,200 and 1,600 from the anterior extremity (Figures 1A, 2A). Haptor 1,250-1,400 (1,325) long by 1,250-1,675 (1,508) wide. Six haptoral suckers, cup-shaped with elaborate skeletal elements, 225-440 (412; n = 18) long by 315-465 (349; n = 18) wide (Figures 1A, 2A, 3B). Egg 250 and 275 long by 200 and 275 wide. Hamuli absent. Marginal hooklets 13-15 (14; n = 3) long retained in adult parasites, similar in size and shape (Figure 1C).

### Remarks

The new species is similar to *Polystomoides* species previously assigned to *Neopolystoma*, considering the absence of hamuli: *P. aspidonectis* (MacCallum, 1918), *P. cayensis* (Du Preez et al., 2017), *P. cyclovitellum* (Caballero et al., 1956), *P. domitilae* (Caballero, 1938), *P. euzeti* (Combes & Ktari, 1976), *P. exhamatum* (Ozaki, 1935), *P. orbiculare* (Stunkard, 1916), *P. rugosa* (MacCallum, 1918), and *P. terrapenis* (Harwood, 1932).

The new species is similar to *P. cyclovitellum*, *P. exhamatum*, *P. orbiculare*, *P. terrapenis* and *P. cayensis*, in terms of the number of spines in the genital atrium. *Polystomoides santamariensis* n. sp. differs from these congeneric species on the length of the genital spines, which are longer (Stunkard, 1916; Harwood, 1932; Ozaki, 1935; Price, 1939; Caballero et al., 1956; Lamothe-Argumedo, 1972; Du Preez et al., 2017). Besides, regarding the number of genital atrium spines and absence of hamuli, it resembles *Fornixtrema fentoni* (Platt, 2000), *Fornixtrema guianensis* (Du Preez et al., 2017), *Fornixtrema scorpioides* (Du Preez et al., 2017), and *Pleurodirotrema chelodinae* (MacCallum, 1918), species previously allocated in *Neopolystoma*, differing also from the size of genital spines.

According to Du Preez et al. (2022), the inter-vaginal distance that corresponds to *Polystomoides* infecting cryptodires ranges from 84.2-99.4% and the intervaginal distance observed in *P. santamariensis* fits this range. Nonetheless, these authors stated that the number of genital spines for *Polystomoides* infecting cryptodires ranges from 24 to 44, which is not in agreement to the number presented in the specimens described herein.

Du Preez & Theunissen (2021) proposed four types of haptoral suckers for polystomes. Their Type III corresponds to Type 2 of Pichelin (1995) and is the type present in all polystomes infecting chelonian hosts. These suckers are spherical, symmetrical, firm, directed ventrolaterally and characterized by having embedded skeletal elements that provide a secure grip on the host tissue. The morphology of the haptoral suckers of *Polystomoides santamariensis* n. sp. is in agreement with type III proposed by these authors.

## Discussion

*Polystomoides* was originally described as a sugbgenus of *Polystoma* Zeder, 1800 by Ward (1917), based on the presence of a short ovary with a single egg, being elevated to genus rank by Ozaki (1935) who pointed to the absence of a uterus as a diagnostic character (Chaabane et al., 2022). Species of the genus are found in the mouth, esophagus, nasal cavities and urinary bladder of turtles and had been charaterized by the presence of two pairs of hamuli in the haptor (Vieira et al., 2008; Chaabane et al., 2022). Price (1939) proposed two new genera for polystomatids of chelonians: *Polystomoidella* Price, 1939 parasites of the urinary bladder of their hosts and *Neopolystoma* Price, 1939 found in the urinary bladder and nasal cavities, differing in the absence and number of hamuli, *Polystomoides* spp. have 2 pair of hamuli, *Polystomoidella* spp. have 1 pair and *Neopolystoma* spp. none (Vieira et al., 2008; Du Preez & Van Rooyen, 2015; Du Preez & Verneau, 2020; Chaabane et al., 2022). Only recently, the polystomatid genera parasitizing chelonians received considerable attention, with the description of *Uropolystomoides*; *Uteropolystomoides*; *Aussietrema*; *Fornixtrema*; *Apaloneotrema*; *Manotrema* and *Pleurodirotrema*; and the synonymizing of *Neopolystoma* to a junior synonym of *Polystomoides* [see Tinsley (2017); Tinsley & Tinsley (2016); Du Preez & Verneau (2020); Du Preez et al. (2022)]. Some species previously allocated in *Polystomoides* were transferred to *Uropolystomoides*, *Uteropolystomoides*, and *Manotrema* based in one or more characteristics (Du Preez et al., 2022; Price, 1939; Tinsley, 2017; Tinsley & Tinsley, 2016). All the South American species of *Polystomoides* infecting the oral region Pleurodira freshwater turtles were transferred to *Manotrema: Manotrema uruguayensis* (Mané-Garzón & Gil, 1961), *Manotrema fuquesi* (Mané-Garzón & Gil, 1962) and *Manotrema brasiliensis* (Vieira et al., 2008) (Du Preez et al., 2022).

Chaabane et al. (2022) proposed the name *Polystomoides* for the clade composed by *Neopolystoma* and *Polystomoides*, considering the similarity in the morphology of the vaginae, reassigning nine species, previously attributed to *Neopolystoma*, for *Polystomoides*, and redefined the genus as polystomes species infecting oral cavity and urinary bladder of cryptodires, presenting or not hamuli and peripheral vaginae. Based on its unique morphology they retained *Uteropolystomoides* although it shares the same clade (Chaabane et al., 2022). Considering this proposition, the new species is allocated in *Polystomoides*, being the first species of the genus described in Brazil.

Considering the recent studies regarding the taxonomy of the polystomes parasites of turtles, further molecular studies are needed to clarify the phylogenetic relationships of these genera, to be compared to morphological and biological findings. Polystomes are grouped mainly by the site of infection considering that they present site specificity, which can lead to the process of speciation and explain the diversity of parasite species found in freshwater turtles (Du Preez & Van Rooyen, 2015). Given the enormous diversity of freshwater turtles around the world, it is likely that a large number of chelonian polystomatids are still unknown.

The present paper highlighted the value of non-invasive and non-lethal procedures to investigate the polystomatid fauna since the specimens were collected during surgery.
